# Microstructural Analysis of Peripheral Lung Tissue through CPMG Inter-Echo Time R2 Dispersion

**DOI:** 10.1371/journal.pone.0141894

**Published:** 2015-11-06

**Authors:** Felix T. Kurz, Thomas Kampf, Lukas R. Buschle, Heinz-Peter Schlemmer, Sabine Heiland, Martin Bendszus, Christian H. Ziener

**Affiliations:** 1 Department of Neuroradiology, Heidelberg University, Heidelberg, Germany; 2 Department of Radiology, German Cancer Research Center, Heidelberg, Germany; 3 Department of Experimental Physics 5, Würzburg University, Würzburg, Germany; University Hospital of Würzburg, GERMANY

## Abstract

Since changes in lung microstructure are important indicators for (early stage) lung pathology, there is a need for quantifiable information of diagnostically challenging cases in a clinical setting, e.g. to evaluate early emphysematous changes in peripheral lung tissue. Considering alveoli as spherical air-spaces surrounded by a thin film of lung tissue allows deriving an expression for Carr-Purcell-Meiboom-Gill transverse relaxation rates *R*_2_ with a dependence on inter-echo time, local air-tissue volume fraction, diffusion coefficient and alveolar diameter, within a weak field approximation. The model relaxation rate exhibits the same hyperbolic tangent dependency as seen in the Luz-Meiboom model and limiting cases agree with Brooks et al. and Jensen et al. In addition, the model is tested against experimental data for passively deflated rat lungs: the resulting mean alveolar radius of *R*_A_ = 31.46 ± 13.15 *μ*m is very close to the literature value (∼34 *μ*m). Also, modeled radii obtained from relaxometer measurements of ageing hydrogel foam (that mimics peripheral lung tissue) are in good agreement with those obtained from *μ*CT images of the same foam (mean relative error: 0.06 ± 0.01). The model’s ability to determine the alveolar radius and/or air volume fraction will be useful in quantifying peripheral lung microstructure.

## Introduction

Structural and functional changes in pulmonary disease are generally tightly linked to alterations in lung microstructure, most familiar in pulmonary emphysema, where remodeling and/or obliteration of small acini and alveoli, as well as parenchymal tissue destruction, lead to an increasing obstruction of the lung’s airways [1, 2]. Pulmonary function tests are not able to separate between different forms of the underlying tissue pathology, especially when tissue alterations are heterogeneously distributed throughout the entire lung as in early stage emphysema [3, 4]. Consequently, there is a need for quantitative lung imaging to assess the extent of microstructural changes and gain a deeper understanding of the associated pathophysiological processes. Advances in imaging technology have been made by introducing high-resolution computed tomography [5, 6] (HRCT), yet microscopic structures, such as acini and alveoli cannot be resolved in detail in HRCT and, therefore, *ex vivo* histopathological analyses through lung stereology are still required to accurately evaluate the extent of emphysematous changes in lung microstructure [7]. Recently, however, there has been a growing interest in magnetic resonance imaging of pulmonary microstructral alterations due to the development of novel imaging techniques and contrast agents [8–11].

For instance, Yablonskiy *et al*. proposed an imaging technique based on a model of cylindrical acinar airways that are covered by alveoli to provide quantitative information on lung morphometry while measuring the diffusivity of inhaled hyperpolarized ^3^He gas [12, 13]. Other models consider lung tissue as a collection of air-filled spherical shells or spherical, cubical or polyhedral (Wigner-Seitz) air spaces, also coined “foam” models, in a medium that consists mostly of water and/or blood [14–19]. The current study will make use of the alveolar Wigner-Seitz foam model because of its mathematical simplicity and since recent results have been shown to be very similar to those obtained from more intricate models [15, 20].

Due to macroscopic susceptibility shifts and differences between lung and mediastinal tissue, gradient-echo based sequences are rarely applicable in the clinical setting. However, macroscopic susceptibility gradients and the corresponding signal distortions can be reduced to a minimum by applying spin-echo sequences or their extension in terms of a multi-spin-echo Carr-Purcell-Meiboom-Gill (CPMG) sequence. Dephasing of transverse magnetization due to the magnetic field inhomogeneities at boundary surfaces of intrapulmonary air and liquid or solid tissue are refocusable through the use of spin echoes. Refocusing, however, is limited by diffusion effects within the tissue. CPMG sequences consist of a 90° pulse followed by a train of equidistant 180° pulses and have been shown to decrease the effect of diffusion on spin dephasing [21, 22]. Relaxation rates in CPMG experiments can then be analyzed by varying the respective inter-echo time.

Experimental studies showing the dependence of the CPMG relaxation rate on the pulse sequence inter-echo time of lung tissue were first performed by Shioya *et al*. [23]. They examined both passively deflated and degassed lung tissue in male Wistar rats and found slow and fast components in terms of a biexponential decay. It was postulated that there is a dependency of inter-echo time on fast and slow *T*_2_ components that might be attributable to different diffusion constants in the lung in accordance with Laicher *et al*. [24]. Another set of experiments has been performed by Baete *et al*. [19], who examined the dependence of relaxation rates on inter-echo times in hydrogel foams to obtain the foam’s underlying microstructural parameters. Hydrogel foams, like lung tissue, can be considered as a porous medium: they are biphasic systems that consist of air bubbles separated by thin layers of hydrogel [25]. Baete *et al*. could show, through X-ray micro-CT imaging, that hydrogel foams nicely mimic lung tissue.

Recently, a weak field approximation was introduced by Jensen and Chandra to examine weak local susceptibility differences and their influence on NMR relaxation rate [26]. The approximation describes local field inhomogeneities through dipole fields to consider diffusion effects and thereby utilizes a frequency correlation function that is tightly linked to the dephasing process. It incorporates microscopic tissue parameters such as the local volume fraction of magnetic perturbers, the diffusion coefficient and the size of the inhomogeneity [27, 28]. While, within this context, CPMG signal formation was recently investigated by Ziener *et al*. [29], the study at hand extends and furthens this previous analysis by examining relaxation rates through general (Fourier) boundary conditions and provides new and simpler expressions for the relaxation rate and associated coefficients by using novel analytical techniques [30] to methodologically investigate CPMG signal formation and its relation to microstructural parameters of lung parenchyma and lung-tissue-like hydrogel foams.

## Methods

### General theory

**Fig 1:**
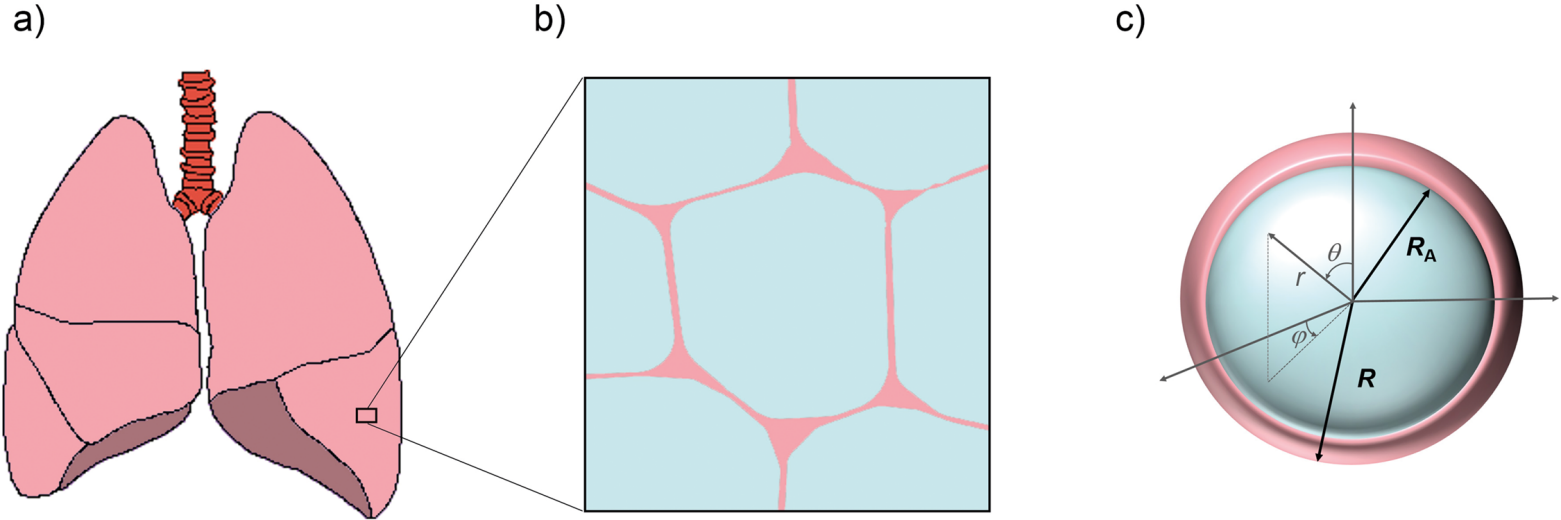
Schematic view of peripheral lung tissue and model geometry. From the left lower pulmonary lobe of the human lungs (a), a lung tissue segment with neighboring alveoli (b) is enlarged: the center alveolus is shown as the cross-section of a rhombic dodecahedron or Wigner-Seitz cell, with thin films as tissue walls (see main text for details and [15]). (c) Schematic cross section of a single alveolus in spherical form with alveolar radius *R*_A_, radius of the dephasing volume *R* and a set of spherical coordinates (*r*, *θ*, *ϕ*).

The Wigner-Seitz foam model for peripheral lung tissue is based on the notion of alveoli as rhombic dodecahedral air-spaces embedded in a surrounding medium [15] (see also Fig 1a and 1b). This allows for volume fractions of air content to be considered close to 1 as opposed to simple spherical foam-models, where volume fractions do not exceed the upper limit for close-packed spheres at 0.74. In further approximation, both the dodecahedral air volume and its surrounding dodecahedral volume are replaced by that of a sphere of radius *R*_A_ and *R*, respectively, such that the local volume fraction $\eta =R_{\text{A}}^{3}/R^{3}$ (see Fig 1c), in analogy to [31]. The surrounding sphere volume is a mathematical construct based on the volume of the Wigner-Seitz-cell and does not reflect the actual anatomical conditions. However, in relation to the internal sphere volume it produces the correct local air volume fraction which is a direct measure of local lung air content. It should be noted that *η* is not a measure of the mean alveolar diameter since the radius *R* of the alveolus-surrounding sphere is generally unknown.

In an external magnetic field, *B*_0_, three-dimensional dipole fields are generated around the alveoli [16]. As in previously established models, the influence of interaction effects between the magnetic fields of neighboring alveoli is neglected [15, 20]; one reason to favor this simplification of the mathematical approach is its good results in reproducing the experimental NMR-lineshape for the free induction decay [15]. Consequently, diffusion-dependent proton spin movement around an alveolus is considered to be restricted to a shell-like dephasing volume $V=\frac{4}{3}\pi \left[R^{3}-R_{\text{A}}^{3}\right]$.

In spherical coordinates **r** = (*r*, *θ*, *ϕ*) (Fig 1c), the local spin resonance frequency *ω*(**r**) depends on *r* and *θ* only

$$\begin{array}{c} \omega \left(r\right)=\omega \left(r,\theta \right)=\delta \omega R_{\text{A}}^{3}\frac{3\cos^{2}\left(\theta \right)-1}{r^{3}}\; \end{array}$$ (1)

where prefactor *δω* = *γB*_0_ Δχ/3 is the equatorial frequency shift that characterizes the strength of the magnetic field distortion, proton gyromagnetic ratio *γ* = 2.675 × 10^8^ rad s^−1^T^−1^ and susceptibility difference Δχ. Stochastic spin fluctuations of water protons in the external *B*_0_ field are described by allocation of a spin transition probability *p*(**r**,**r**_0_, *t*) that accounts for the probability that a spin in position **r**_0_ diffuses to position **r** in time *t*. The probability function *p*(**r**,**r**_0_, *t*) can be obtained by solving the diffusion equation [27]

$$\begin{array}{c} \frac{\partial }{\partial t}p\left(r,r_{0},t\right)=D\Delta p\left(r,r_{0},t\right)\; \end{array}$$ (2)

with diffusion coefficient *D*, and *p*(**r**,**r**_0_, *t*) = *e* ^*tD*Δ^ *δ*(**r** − **r**_0_). It is advantageous to perform a spectral expansion of *p*(**r**,**r**_0_, *t*) as

$$\begin{array}{c} p\left(r,r_{0},t\right)=\sum_{n}e^{-\kappa_{n}^{2}\frac{t}{\tau }}\psi_{n}\left(r\right)\psi_{n}\left(r_{0}\right)\; \end{array}$$ (3)

to solve Eq (2). The eigenfunctions *ψ*_*n*_(**r**) thereby obey

$$\begin{array}{c} \Delta \psi_{n}\left(r\right)=-\frac{\kappa_{n}^{2}}{D\tau }\psi_{n}\left(r\right)\; \end{array}$$ (4)

and *τ* represents the characteristic time as a measure of diffusion:

$$\begin{array}{c} \tau =\frac{R_{\text{A}}^{2}}{D}\;. \end{array}$$ (5)

Furthermore, a frequency correlation function *K*(*t*) can be introduced that is a measure of spin fluctuations induced by the local magnetic field inhomogeneity in the dephasing volume *V* (see also [26, 32]):

$$\begin{array}{ccc} K(t) & = & \frac{1}{V}\int_{V}\;d^{3}r\;\int_{V}\;d^{3}r_{0}\omega \left(r\right)p\left(r,r_{0},t\right)\omega \left(r_{0}\right) \end{array}$$ (6)

$$\begin{array}{ccc} & = & \frac{1}{V}\int_{V}\;d^{3}r\;\omega \left(r\right)e^{tD\Delta }\omega \left(r\right) \end{array}$$ (7)

$$\begin{array}{ccc} & = & \delta \omega^{2}\sum_{n=1}^{\infty }G_{n}e^{-\kappa_{n}^{2}\frac{t}{\tau }}\;, \end{array}$$ (8)

and the expansion coefficients *G*_*n*_ can be obtained through

$$\begin{array}{c} \sqrt{G_{n}}=\frac{1}{\delta \omega \sqrt{V}}\int_{V}\;d^{3}r\omega \left(r\right)\psi_{n}\left(r\right)\;. \end{array}$$ (9)

Within the mean field theory of Anderson and Weiss [33], the relation between correlation function *K*(*t*) and gradient echo signal intensity can be stated as

$$\begin{array}{c} M\left(t\right)=\text{exp}(-\int_{0}^{t}\;\text{d}\xi \left[\xi -t\right]K\left(\xi \right))\;, \end{array}$$ (10)

provided the conditional transition probability between distinct frequencies is Gaussian [32].

Generally, transverse relaxation rate *R*_2_ can be treated as the sum of an intrinsic relaxation rate *R*_2,0_ and a diffusion-related relaxation rate Δ*R*_2_. Then, diffusion-related relaxation rate, Δ*R*_2_, may be expressed as:

$$\begin{array}{ccc} \Delta R_{2} & = & \frac{8}{\pi^{2}}\sum_{m=0}^{\infty }\frac{1}{{\left[2m+1\right]}^{2}}\int_{0}^{\infty }\;dtK\left(t\right)\cos(\left[2m+1\right]\pi \frac{t}{\tau_{180}}) \end{array}$$ (11)

$$\begin{array}{ccc} & = & \frac{8}{\pi^{2}}\sum_{m=0}^{\infty }\frac{1}{{\left[2m+1\right]}^{2}}\sum_{n=1}^{\infty }\frac{\tau \delta \omega^{2}G_{n}\kappa_{n}^{2}}{\kappa_{n}^{4}+{\left[\pi \left[2m+1\right]\tau /\tau_{180}\right]}^{2}}\;, \end{array}$$ (12)

(c.f. Eq (19) in [26]), which is within Jensen and Chandra’s weak field approximation [34] in close analogy to [29] in terms of inter-echo time *τ*_180_, characteristic time *τ* and field-induced susceptibility-dependent frequency shift *δω*.

### Boundary conditions

Water molecules can either be reflected at or move through the alveolar tissue-air interface. Thus, the main mechanism of MR signal decay in peripheral lung tissue is defined by an adequate choice of the surface boundary conditions. General boundary conditions for the eigenfunctions *ψ*_*n*_(**r**) at the alveolar surfaces with radii *R* and *R*_A_ (*R* > *R*_A_) are provided in the form of Fourier boundary conditions [35, 36]:

$$\begin{array}{c} {D\frac{\partial \psi_{n}\left(r,\theta ,\phi \right)}{\partial r}|}_{r=R,R_{\text{A}}}={\rho \;\psi_{n}\left(r,\theta ,\phi \right)|}_{r=R,R_{\text{A}}}\;. \end{array}$$ (13)

These relaxing boundary conditions provide a measure of the surface permeability for water molecules in form of the transfer rate *ρ* (also called surface relaxivity or interface permeability constant) [36]. The following orthogonal eigenfunctions

$$\begin{array}{ccc} \psi_{n}\left(r,\theta ,\phi \right) & = & \frac{3\cos^{2}\left(\theta \right)-1}{M_{n}}\\ & \times & [[y_{2}^{\prime }\left(\kappa_{n}\right)-\frac{\rho R_{\text{A}}}{D\kappa_{n}}y_{2}\left(\kappa_{n}\right)]j_{2}(\frac{\kappa_{n}r}{R_{\text{A}}})-[j_{2}^{\prime }\left(\kappa_{n}\right)-\frac{\rho R_{\text{A}}}{D\kappa_{n}}j_{2}\left(\kappa_{n}\right)]y_{2}(\frac{\kappa_{n}r}{R_{\text{A}}})]\; \end{array}$$ (14)

satisfy the respective boundary condition at *r* = *R*_A_ (with spherical Bessel functions *j*_2_ and *y*_2_ of the first and second kind, respectively, and normalization constant *M*_*n*_). Consequently, the second boundary condition at *r* = *R* leads to the conditional equation

$$\begin{array}{ccc} & & \left[y_{2}^{\prime }\left(\kappa_{n}\right)-\frac{\rho R_{\text{A}}}{D\kappa_{n}}y_{2}\left(\kappa_{n}\right)][j_{2}^{\prime }(\frac{\kappa_{n}}{\sqrt[{3}]{\eta }})-\frac{\rho R_{\text{A}}}{D\kappa_{n}}j_{2}(\frac{\kappa_{n}}{\sqrt[{3}]{\eta }})\right]\\ & & \;=[j_{2}^{\prime }\left(\kappa_{n}\right)-\frac{\rho R_{\text{A}}}{D\kappa_{n}}j_{2}\left(\kappa_{n}\right)][y_{2}^{\prime }(\frac{\kappa_{n}}{\sqrt[{3}]{\eta }})-\frac{\rho R_{\text{A}}}{D\kappa_{n}}y_{2}(\frac{\kappa_{n}}{\sqrt[{3}]{\eta }})]\;. \end{array}$$ (15)

This equation can be solved numerically to obtain the eigenvalues *κ*_*n*_. For impermeable surfaces with a lack of any magnetic impurities that could lead to a vanishing surface relaxation (*ρ* = 0), Eq (13) reduces to Neumann boundary conditions that correspond to reflecting boundaries which are used in [29]. Then, Eq (15) is equivalent to Eq (38) in [27] or Eq (6) in [29].

### Statistics

Fitting routines were performed with the nlm function in MATHEMATICA^®^ (Wolfram Research, Inc., Champaign, IL, USA, [37]).

## Results

### Model properties

#### Eigenvalues and expansion coefficients

**Fig 2:**
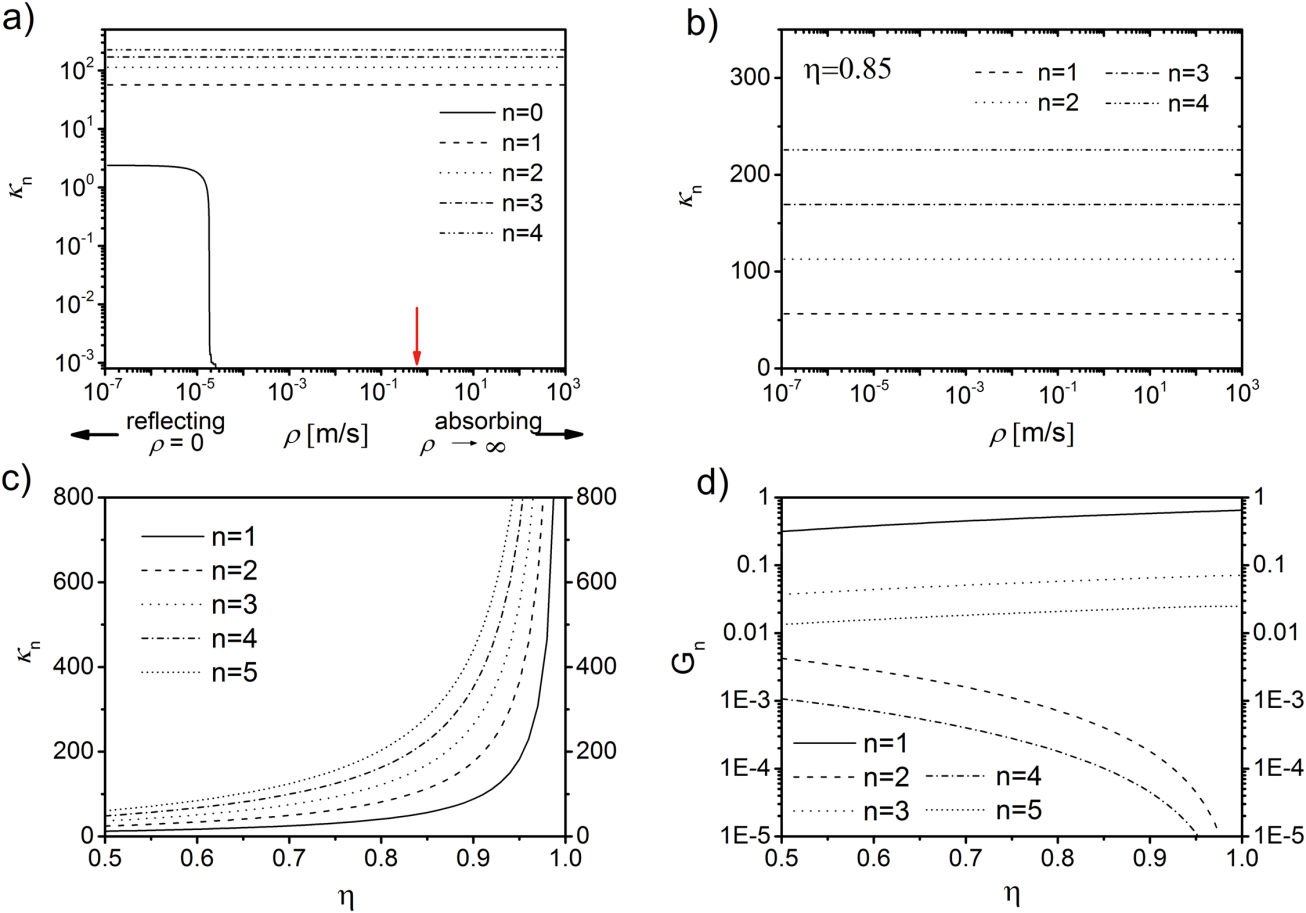
Eigenvalues and expansion coefficients. (a) Lowest eigenvalues, obtained from Eq (15), as a function of surface permeability *ρ*. The red arrow marks the typical surface permeability for peripheral lung tissue *ρ*_L_ ≈ 0.6 [43] (*R*_A_ = 200 *μ*m [40], *D* = 2.3 ⋅ 10^−9^ m^2^s^−1^ [41], *η* = 0.85 [42]). The *ρ*-values of the decisive decrease of the lowest eigenvalue are several orders of magnitude lower than *ρ*_L_. (b) Eigenvalue spectrum for *n* ≥ 1 for the same parameters as in (a). The eigenvalues remain constant over the range of surface permeabilities *ρ*, thus, the assumption of absorbing boundary conditions *ρ* ≈ *ρ*_L_ imposes no significant constraint on the remaining eigenvalue spectrum. (c) Eigenvalues *κ*_*n*_ for absorbing boundary conditions as a function of volume fraction *η*. In the limit *η* → 1, the first eigenvalue *κ*_1_ approaches $\frac{3\sqrt{6}}{1-\eta }$ (see Eq (33)). (d) Expansion coefficients *G*_*n*_ from Eq (9). For *η* → 1, the first expansion coefficient takes the value *G*_1_ ≈ 0.7 and it can be verified that $\sum_{n}G_{n}=\frac{4}{5}$.

For larger volume fractions (*η* > 0.5) or large surface area of alveolar air content when compared to alveolar wall thickness, surface relaxation is not negligible [19]. Specifically, the passage of water molecules through the tissue-air boundary leads to a net loss of water through the respiration process (the partial volume of water vapor in expired air is about six times higher than that in inspired air [38]). High permeability of the alveolar epithelium is ensured by an abundance of aquaporin channels, surfactant and, as has been shown recently, by membrane invaginations consisting of highly water-permeable caveolin proteins [39]. The effect of increasing surface permeability *ρ* on the eigenvalues is demonstrated in Fig 2a for typical parameters of pulmonary tissue (*R*_A_ = 200 *μ*m [40], *D* = 2.3 ⋅ 10^−9^ m^2^s^−1^ [41] and *η* = 0.85 [42]). For very small values of *ρ*, the lowest eigenvalue *κ*_0_ approaches its (finite) limit value for reflecting boundary conditions. However, for increasing values of *ρ*, the lowest eigenvalue quickly descends towards zero, whereas the subsequent eigenvalues remain constant (see Fig 2a and 2b). The region of fast descent is several orders of magnitude lower than the surface relaxivity for pulmonary tissue, *ρ*_L_ ≈ 0.6 ms^−1^ [43] (marked with a red arrow in Fig 2a). Yet, at *ρ* ≈ *ρ*_L_, the eigenvalue spectrum approximately coincides with that of *ρ* → ∞ (Fig 2b). Therefore, absorbing (or Smoluchowski) boundary conditions are assumed [44] and are equivalent to setting *ψ*_*n*_(*R*_A_) = *ψ*_*n*_(*R*) = 0. In analogy to [27], the eigenfunctions *ψ*_*n*_(*r*, *θ*, *ϕ*) that fulfill Eq (4) can be obtained as

$$\begin{array}{c} \psi_{n}\left(r,\theta ,\phi \right)=\frac{3\cos^{2}\left(\theta \right)-1}{N_{n}}[y_{2}(\frac{\kappa_{n}}{\sqrt[{3}]{\eta }})j_{2}(\kappa_{n}\frac{r}{R_{\text{A}}})-j_{2}(\frac{\kappa_{n}}{\sqrt[{3}]{\eta }})y_{2}(\kappa_{n}\frac{r}{R_{\text{A}}})]\;, \end{array}$$ (16)

with normalization constant *N*_*n*_. Consequently, the expansion parameters, *κ*_*n*_, have to satisfy the eigenvalue Eq (15) in the limit *ρ* → ∞, and we find

$$\begin{array}{c} j_{2}\left(\kappa_{n}\right)y_{2}(\frac{\kappa_{n}}{\sqrt[{3}]{\eta }})=j_{2}(\frac{\kappa_{n}}{\sqrt[{3}]{\eta }})y_{2}\left(\kappa_{n}\right)\;. \end{array}$$ (17)

This transcendental equation has to be solved numerically; for large *η*, the eigenvalues approach infinity with eigenvalue *κ*_1_ ascending the slowest as shown in Fig 2c. The first eigenvalue, *κ*_1_, can be approximated with Eq (17) as

$$\begin{array}{c} \kappa_{1}\approx \frac{\sqrt{42}\eta^{\frac{1}{3}}}{1-\eta^{\frac{1}{3}}}\sqrt{\frac{1+\eta^{\frac{1}{3}}+\eta^{\frac{2}{3}}+\eta +\eta^{\frac{4}{3}}}{3+9\eta^{\frac{1}{3}}+11\eta^{\frac{2}{3}}+9\eta +3\eta^{\frac{4}{3}}}}\;. \end{array}$$ (18)

In addition, by solving Eq (9) with Eqs (16) and (1), and using analytical techniques from [30], the dimensionless expansion coefficients, *G*_*n*_, are given as:

$$\begin{array}{c} G_{n}=\frac{24\eta }{5\left[1-\eta \right]\kappa_{n}^{2}}\;\frac{{\left[j_{2}\left(\kappa_{n}\eta^{-\frac{1}{3}}\right)-\eta j_{2}\left(\kappa_{n}\right)\right]}^{2}}{\eta^{\frac{1}{3}}{\left[j_{2}\left(\kappa_{n}\right)\right]}^{2}-{\left[j_{2}\left(\kappa_{n}\eta^{-\frac{1}{3}}\right)\right]}^{2}}\;. \end{array}$$ (19)

An expression for *G*_*n*_ in terms of trigonometric functions is provided in Eq (28) in Appendix A. Since the eigenvalues *κ*_*n*_ depend on the volume fraction *η* only, the same dependence holds for the expansion coefficients *G*_*n*_ and is visualized in Fig 2d. Naturally, $\sum_{n}G_{n}=K\left(0\right)/\delta \omega^{2}=\langle \omega^{2}\left(r\right)\rangle /\delta \omega^{2}=\frac{4}{5}\eta$, where we have used Eqs (1), (7) and (8). This corresponds to Eq (18) in [45] for the local frequency variance. Further sums over combinations of *G*_*n*_ and *κ*_*n*_ are helpful to estimate the number of addends in the infinite sums with sufficient accuracy. They are provided in Appendix A.

#### Model relaxation rate and correlation time

**Fig 3:**
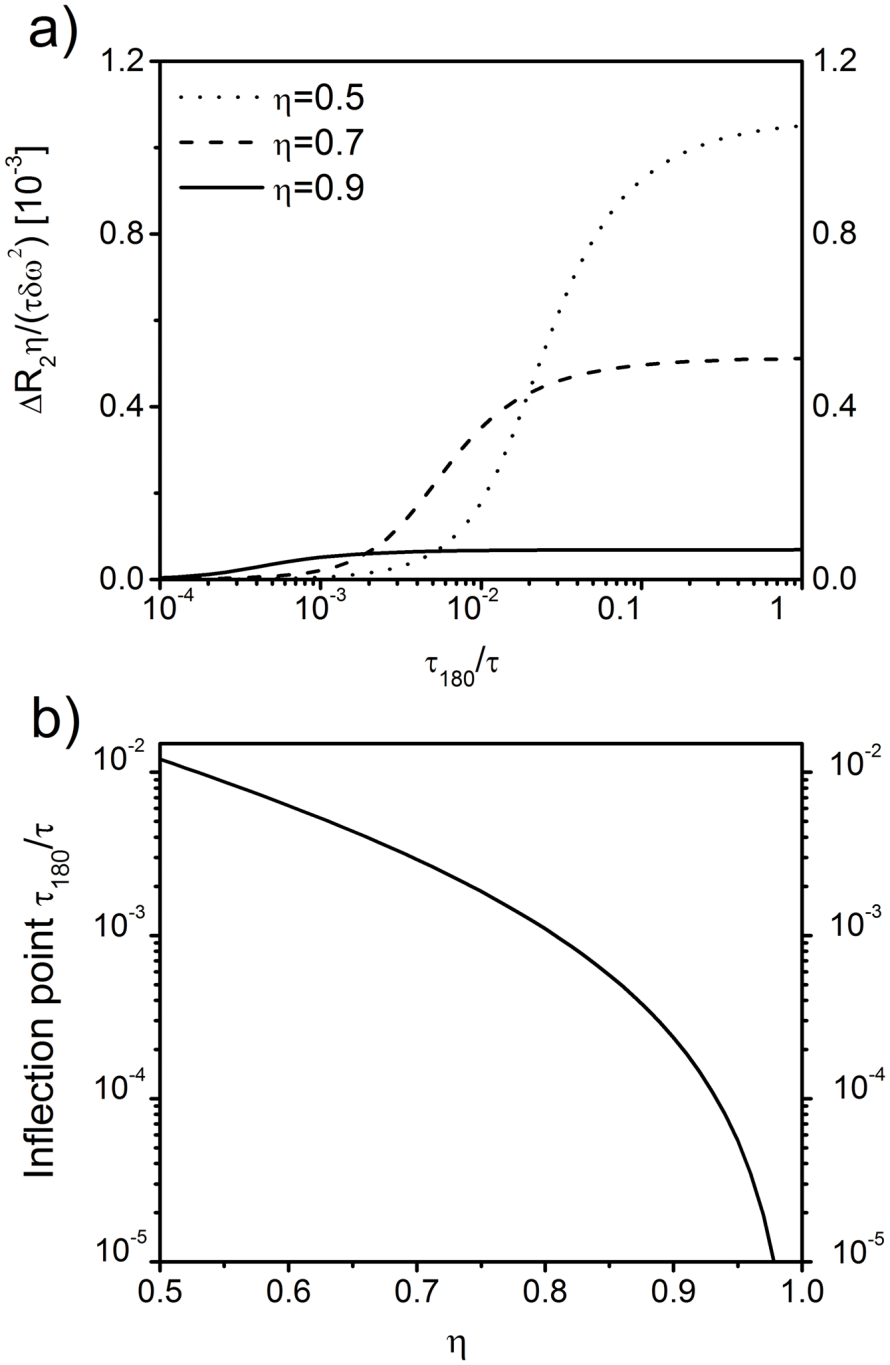
CPMG inter-echo relaxation rate dispersion. (a) Dependence of CPMG relaxation rate Δ*R*_2_ on the inter-echo time and volume fraction as obtained from Eq (20). (b) Values of *τ*_180_/*τ* at the inflection points of the Δ*R*_2_ relaxation rate curve for different regional blood volumes fractions *η*. For *η* = 0.8, the inflection point possesses a value of *τ*_180_/*τ* = 0.001.

The diffusion-related relaxation rate, Δ*R*_2_ in Eq (12), can be transformed to

$$\begin{array}{c} \frac{\Delta R_{2}}{\tau \delta \omega^{2}}=\sum_{n=1}^{\infty }\frac{G_{n}}{\kappa_{n}^{2}}[1-\frac{2\tau }{\kappa_{n}^{2}\tau_{180}}\tanh(\frac{\kappa_{n}^{2}\tau_{180}}{2\tau })]\;, \end{array}$$ (20)

which is in agreement with general scaling properties of transverse relaxation times [46]. The hyperbolic tangent dependency on *τ*_180_ of Δ*R*_2_ corresponds to the Luz-Meiboom model [47]. In Fig 3a, Δ*R*_2_ is visualized as a function of normalized inter-echo time *τ*_180_/*τ* for three different volume fractions. For increasing inter-echo time, Δ*R*_2_ reaches a plateau whose value depends on the limit of Eq (20) for *η* → 1. In this limit, only the eigenvalue *κ*_1_ significantly contributes to the correlation function *K*(*t*) (see Fig 2a). The correlation function *K*(*t*) then decays mono-exponentially as $K\left(t\right)=\delta \omega^{2}G_{1}\exp\left(-\kappa_{1}^{2}t/\tau \right)\approx 0.7\;\delta \omega^{2}\exp\left(-\kappa_{1}^{2}t/\tau \right)$, c.f. Fig 2b.

The correlation time *τ*_C_ follows from the mean relaxation time approximation [48]

$$\begin{array}{c} \tau_{\text{C}}=\int_{0}^{\infty }\frac{K(t)}{K(0)}\text{d}t=\frac{5\tau }{4\eta }\sum_{n=1}^{\infty }\frac{G_{n}}{\kappa_{n}^{2}} \end{array}$$ (21)

$$\begin{array}{c} =\frac{5\tau }{384}\frac{{\left[\eta^{\frac{1}{3}}-1\right]}^{2}\left[4+7\eta^{\frac{1}{3}}+4\eta^{\frac{2}{3}}\right]}{\left[1+\eta^{\frac{1}{3}}+\eta^{\frac{2}{3}}\right]\left[1+\eta^{\frac{1}{3}}+\eta^{\frac{2}{3}}+\eta +\eta^{\frac{4}{3}}\right]}\;. \end{array}$$ (22)

where $\lim_{x\to \infty }\frac{\tanh(x)}{x}=0$ from Eq (20) and the evaluation of the sum is provided in Eq (31) in Appendix A. With this expression for *τ*_C_, we can rewrite the model relaxation rate from Eq (20) as:

$$\begin{array}{c} \Delta R_{2}=\frac{4}{5}\eta \tau_{\text{C}}\delta \omega^{2}-\frac{2{\left[\tau \delta \omega \right]}^{2}}{\tau_{180}}\sum_{n=1}^{\infty }\frac{G_{n}}{\kappa_{n}^{4}}\tanh(\frac{\kappa_{n}^{2}\tau_{180}}{2\tau })\;. \end{array}$$ (23)

Moreover, to provide a starting point for experimental curve sampling, it is reasonable to consider the point where CPMG relaxation rates show the strongest change for alterations of *τ*_180_. Naturally, such a point is given by the inflection point *τ*_180_/*τ* of the curve Δ*R*_2_/[*τδω* ^2^] which describes the intermediate regime of *τ*_180_ close to the characteristic time *τ*. Inflection points were numerically evaluated depending on air volume fraction *η* (see Fig 3b). Typical values of *η* range between 0.5–1.0 for lung tissue. Fig 3b demonstrates that inflection points at *τ*_180_/*τ* decrease exponentially towards zero for *η* → 1. For an air volume fraction of *η* = 0.8, the inflection point is located at *τ*_180_ ≈ 0.001*τ*.

#### Limiting cases

For large inter-echo times, the relaxation rate for the spin echo (and gradient echo) can be obtained with Eq (23) as

$$\begin{array}{c} \lim_{\tau_{180}\to \infty }\Delta R_{2}=\tau \delta \omega^{2}\sum_{n=1}^{\infty }\frac{G_{n}}{\kappa_{n}^{2}}=\tau_{\text{C}}\frac{4}{5}\eta \delta \omega^{2}=\tau_{\text{C}}\left\langle \omega^{2}\left(r\right)\right\rangle \;, \end{array}$$ (24)

in agreement with the well-known motional-narrowing limit, see [26] and footnote 1 in [49].

For *τ*_180_ → 0, the quadratic dependence of CPMG relaxation rate on *τ*_180_ can be calculated with Eq (30) from Appendix A as

$$\begin{array}{c} \lim_{\tau_{180}\to 0}\frac{\Delta R_{2}}{\tau \delta \omega^{2}}=\frac{1}{12}{\left[\frac{\tau_{180}}{\tau }\right]}^{2}\sum_{n=1}^{\infty }G_{n}\kappa_{n}^{2}=\frac{3}{5}{\left[\frac{\tau_{180}}{\tau }\right]}^{2}\eta \frac{1-\eta^{\frac{5}{3}}}{1-\eta }\;, \end{array}$$ (25)

where we have used the fact that $\lim_{x\to 0}\frac{\tanh(x)}{x}=1-\frac{x^{2}}{3}+O\left(x^{3}\right)$. This is in accordance with Eq (15) in [29] and the quadratic dependency of Δ*R*_2_ on inter-echo time *τ*_180_ agrees with Eq (16a) for the short-echo limit in [49].

The case of very large volume fractions, *η* → 1, is of interest for testing numerical accuracy and is briefly discussed in Appendix B.

### Experimental verification

#### Passively deflated lung tissue

**Fig 4:**
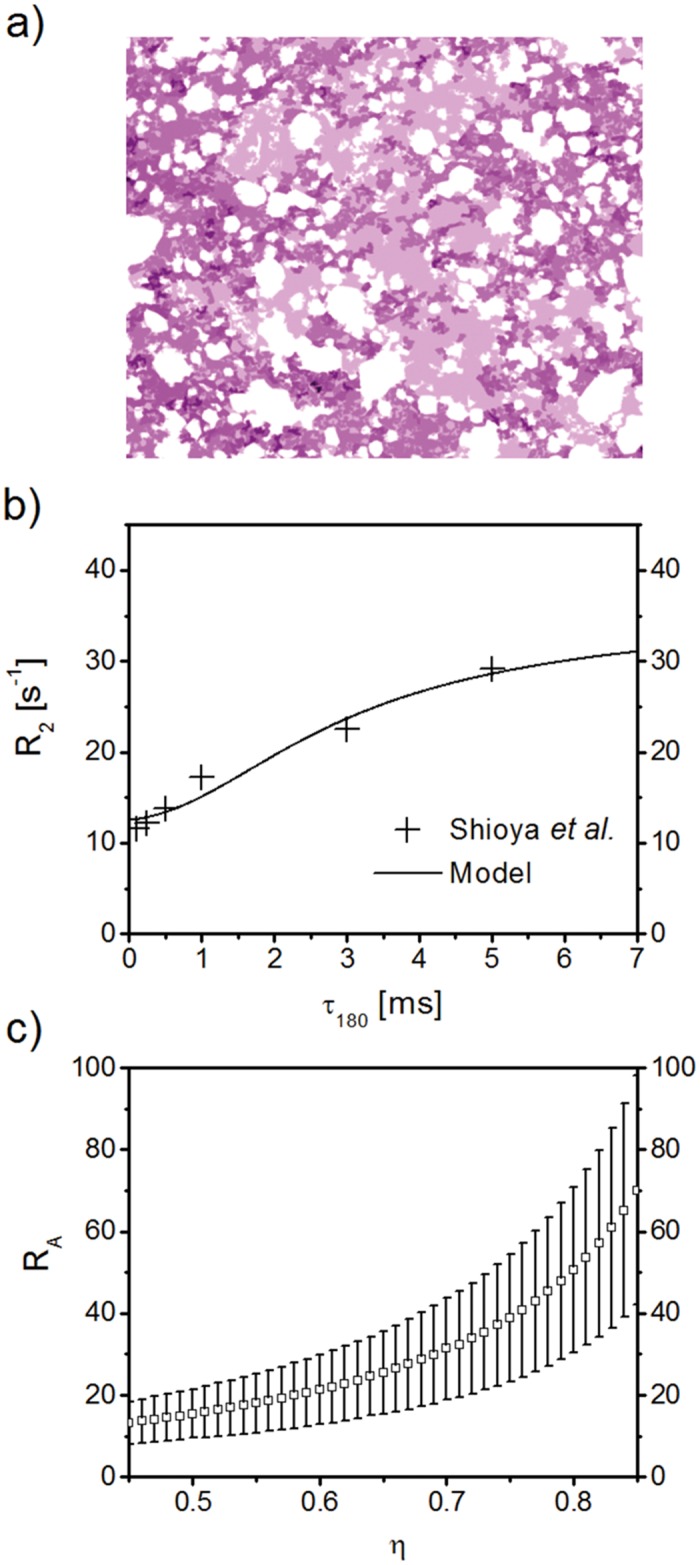
Model CPMG relaxation rate as a function of inter-echo time *τ*_180_. (a) Sketch of passively deflated lung tissue, modified from [23]. Air filled spaces or alveoli for passively deflated lung tissue are less numerous and prominent than in non-deflated lung tissue. (b) Relaxation rate *R*_2_ for passively deflated lung tissue (continuous line) in comparison with experimental data [23]. The analytical model is fitted to the experimental data points, with resulting fitted values of characteristic time *τ* = 0.56 ± 0.22 s (*p* = 0.088) and intrinsic relaxation rate *R*_2,0_ = 12.58 ± 0.96 s^−1^ (*p* = 9.72 ⋅ 10^−4^). With the use of Eq (5), the mean alveolar radius follows as *R*_A_ = 31.46 ± 13.15 *μ*m, which is in very good agreement with the expected value of ∼34 *μ*m [41, 51]. (c) Model mean alveolar radius *R*_A_ for different air volume fractions *η* (error bars represent the standard error of *R*_A_ from the model fit; *p*-values never exceeded 0.088). Naturally, the mean alveolar radius increases with increasing air volume fraction and reaches a value of *R*_A_ = 70.12 ± 28.04 *μ*m for *η* = 0.85.

In Fig 4, theoretical results for Δ*R*_2_ are compared to experimental data for excised peripheral lung samples of Wistar rats as performed by Shioya *et al*. at 2.11 T [23]. For passively deflated lung tissue, the initial lung air volume content in the alveolar region can be assumed as *η*_0_ = 85.4% [40]. Moreover, passively collapsed rat lungs still contain about 40% of their initial air volume [23], thus, the air volume fraction for passively deflated peripheral lung tissue follows as $\frac{0.4\eta_{0}}{1-0.6\eta_{0}}=0.70$, c.f. Fig 4a. Furthermore, the mean alveolar diameter in (non-deflated) rat lungs is given by the mean linear intercept as demonstrated in [50]. Under normal physiological conditions, it usually assumes values of 80–100 *μ*m, yet the value 92 *μ*m as determined in [51] will be used for further calculations. Consequently, the expected value of the alveolar radius is 46 *μ*m in rat lungs (humans: 200 *μ*m [42]). However, if homogeneous shrinkage of the alveoli is assumed, the alveolar radius in peripheral lung tissue can be determined with the above assumptions as $R_{\text{A}}^{\text{E}}=\sqrt[{3}]{0.4}\cdot 46\;\mu m=33.89\;\mu m$. While fitting the model to the experimental parameters, we obtain *τ* = 0.56 ± 0.22 s (*p* = 0.088) and *R*_2,0_ = 12.58 ± 0.96 s^−1^ (*p* = 9.72 ⋅ 10^−4^), see Fig 4b. The correlation time can be achieved with Eq (21) as *τ*_C_ = 1.44 ± 0.58 ms. Naturally, the model relaxation rate curve follows the experimental values with a sigmoidal increase in relaxation rate for increasing inter-echo times. With a typical proton spin diffusion coefficient *D* = 2.3 *μ*m^2^ms^−1^ in lung tissue [41], the mean local alveolar radius as determined with Eq (5) gives *R*_A_ = 31.46 ± 13.15 *μ*m, which is in very good agreement with the expected value $R_{\text{A}}^{\text{E}}$ (see also Appendix C). Fig 4c shows model mean alveolar radius for different air volume fractions *η* (error bars represent the standard error for *R*_A_ from the fitting result): as expected, the mean alveolar radius increases with increasing air volume fraction and reaches a value of *R*_A_ = 70.12 ± 28.04 *μ*m for *η* = 0.85.

#### Hydrogel foam

**Fig 5:**
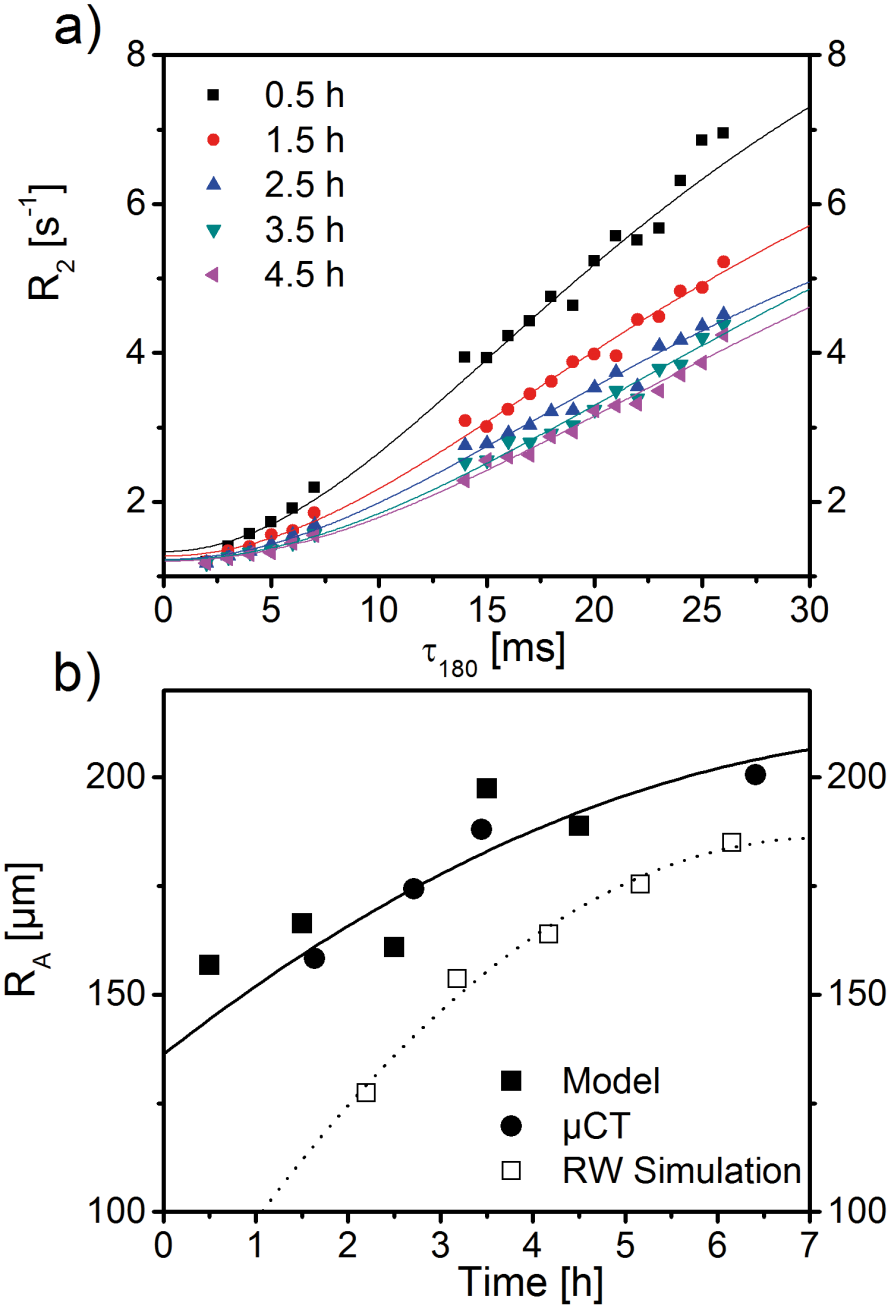
Relaxation rate dispersion and quantification of mean air bubble radius for ageing hydrogel foam. (a) Several experimental *R*_2_ values (symbols) measured at different imaging times of ageing hydrogel foam with a 0.5 *T* benchtop relaxometer [19] was used to fit the analytical model with *R*_2_ from Eq (23) and respective spectral parameters as determined above (solid lines; for further details, please see main text). Fit parameters for characteristic time *τ* and intrinsic relaxation rate *R*_2,0_ can be found in Table 1. (b) Mean air bubble radius as obtained through Eq (5) from the different values for *τ* of the fitted model. These values are compared to values obtained by triangulating *μ*CT images of voxel size 19.4 *μ*m of the same foam cross-sections that served to acquire the *R*_2_ dispersion curves [19]. In addition, the time evolution of radii by random walk simulations is shown as performed in [19]. The continuous lines are fits of second order polynomials to the data. The mean relative error of the model and random walk simulation data points to the fit curve of *μ*CT data is 5.84 ± 1.28% and 14.36 ± 2.66%, respectively.

More detailed measurements for *R*_2_ dispersion at air-water interfaces have been performed by Baete *et al*. who examined the microstructural properties of hydrogel foam with a 0.5 T benchtop relaxometer (Bruker Minispec^TM^ mq20), see Fig 7a in [19]. Such hydrogel foams mimic peripheral lung tissue samples and, therefore, provide an adequate means of probing NMR techniques to evaluate and quantify lung microstructure. Over a period of several hours, measured relaxation rates decrease over time while still maintaining an increase with prolonged CPMG inter-echo intervals *τ*_180_, as can be seen in Fig 5a. This corresponds to a coarsening of the foam where air bubbles grow in size and decrease in number. For a diffusion coefficient *D* = 1.062 *μ*m^2^ms^−1^ and an air volume fraction of *η* = 1/1.1667, as determined by Baete *et al*. [19], model fit parameters for *τ* and *R*_2,0_ are summarized in Table 1. The resulting mean air bubble radii, from Eq (5), at different imaging times of the ageing hydrogel foam are depicted in Fig 5b in comparison with *μ*CT-measurements and random walk simulations from [19]. The *μ*CT-images were obtained from the same cross-section of the gel foam and the mean air bubble radii were calculated from triangulated surfaces in voxels of size 19.4 *μ*m as detailed in [19]. Fig 5b further shows radii of random walk simulations (with *D* and *η* as above) taken from [19]. The continuous curves are simple second-order polynomials *P*, i.e. *P*(*t*) = *at* ^2^ + *bt* + *c*, that are fitted to the data points. For the *μ*CT experimental values, *a* = −0.95 ± 0.28 *μ*mh^−2^, *b* = 16.65 ± 3.66 *μ*mh^−1^ and *c* = 136.29 ± 8.78 *μ*m. The mean relative error of the five model radii points to the *μ*CT curve radii at the same time is 5.84 ± 1.28%, whereas it is 14.36 ± 2.66% for the random walk simulations. Though the fitted curve of the analytical model and that of the random walk simulations are in good agreement with values from the *μ*CT experiment, the analytical model has a smaller mean relative error than the radii obtained from the random walk simulations.

**Table 1:** Fit parameters of intrinsic relaxation rate *R*_2,0_ and characteristic time *τ* in Fig 5a.

Time [h]	*R*_2,0_ [1/s]	SE	*p*-value [10^−9^]	*τ* [ms]	SE	*p*-value [10^−6^]
0.5	1.31	0.11	3.21	7.73	0.88	16.58
1.5	1.27	0.05	0.00	9.20	0.78	0.00
2.5	1.22	0.05	0.00	9.18	0.94	0.04
3.5	1.22	0.05	0.00	11.57	1.5	0.94
4.5	1.20	0.04	0.00	11.55	1.35	0.24

Fit parameters of intrinsic relaxation rate *R*_2,0_ and characteristic time *τ* for fitting Eq (23) to the *R*_2_ dispersion of different imaging times of ageing hydrogel foam as measured with a 0.5 T relaxometer in [19]. SE = Standard Error.

## Discussion

While current models of microstructural quantification of pulmonary tissue focus on diffusion measurements after inhalation of ^3^He gas [12, 13], the work presented here within provides a proof-of-principle concept of a (non-invasive) method to measure lung microstructure without the addends of hyperpolarized noble gas or paramagnetic contrast agent. The model considers lung tissue in a simple model geometry [15] and well-known weak field approximation [34], and connects microstructural parameters such as alveolar radius, diffusion coefficient and local air-tissue volume fraction to the relaxation rate of a CPMG sequence. The obtained model CPMG relaxation rate increases with inter-echo time *τ*_180_, and, for exponential growth of *τ*_180_, follows a sigmoidally shaped curve (see Fig 3). The hyperbolic tangent dependency on *τ*_180_ as in Eq (23) corresponds to that in the Luz-Meiboom two-site exchange model [47] and limiting cases agree with expressions from Brooks *et al*. [49] and Jensen *et al*. [26]. Our analysis is based on results in [29], but goes beyond this previous study by providing new expressions for relaxation rate, expansion coefficients and limiting cases by utilizing general boundary conditions and novel analytical techniques established in [30] for the context of lung tissue imaging, as well as an analysis of the relaxation rate curve inflection.

To analyze the effects of surface permeability on surface relaxation, general (Fourier) boundary conditions were assumed and it was shown, in Fig 2a, that the eigenvalue spectrum at typical parameters for peripheral lung tissue is very close to that of absorbing boundary conditions. The lowest eigenvalue of the diffusion equation, *κ*_0_, quickly approaches zero and, thus, does not contribute to the sum in Eq (23) (again using the fact that $\lim_{x\to 0}\frac{\tanh(x)}{x}=1-\frac{x^{2}}{3}+O\left(x^{3}\right)$). In the opposing limit of vanishing surface permeability, *κ*_0_ approaches the first eigenvalue for reflecting boundary conditions which corresponds to the first obtainable eigenvalue from Eq (38) in [27]. The phenomenon of the existence of an exceptional zero of the defining eigenvalue equation has been studied in detail by Gottlieb [52] and Ziener *et al*. [30]. Another contribution to surface relaxation is caused by the immobilization of proton spins after collision with the tissue-air interface, an effect that is comparable to the accelerated relaxation of hydration layers around proteins [53]. Yet, since the relevant eigenvalues obtained from general and absorbing boundary conditions did not differ significantly for typical lung tissue parameters, the latter were chosen for their computational efficacy.

The incentive to determine the inflection point of the CPMG relaxation rate was to obtain an experimental starting point for curve sampling at strong changes of the relaxation rate. It is shown in Fig 3b that the values of *τ*_180_/*τ* at the inflection points exponentially approach zero for *η* → 1. Generally, approximate knowledge of the mean linear intercept or mean alveolar diameter as well as the typical local diffusion coefficient is sufficient to determine the range of necessary inter-echo times *τ*_180_. However, one should keep in mind that short inter-echo times will be required for the setup of the experiment.

The excellent agreement of model values with experimental data from passively deflated lung tissue [23] and lung phantom (ageing hydrogel foam) measurements [19] support the validity of the model. Yet, some experimental confounders should be mentioned: one confounder might be that the remaining air content in passively deflated lung tissue has been estimated by Shioya to be 40% of the original content whereas this value can be variable: for example, Miura *et al*. found values of 31% [54]. In addition, the passive collapse of lung tissue is not necessarily linked to a homogeneous shrinkage of alveoli. In fact, some alveoli may collapse completely whereas others remain intact. This fact might be reflected in the prominent standard error of the determined radius. Another problem in the experimental setup of the proposed model will be that well-tuned 180° refocusing pulses are hard to accomplish.

An important point in translating the presented model to *in vivo* measurements is the contribution of paramagnetic deoxyhemoglobin in blood vessels to MR signal decay. Generally, the oxygenation levels inside the capillaries rise very quickly from their deoxygenated state to the oxygenated state—in fact, the oxygen partial pressure already climbs 50% of its ascent towards full saturation within about 7% of the capillary length [38]. Therefore, it is possible to assume that the majority of blood in the capillary region is either in or close to the fully oxygenated state, and thus, only has a small susceptibility difference to alveolar water. This susceptibility difference will be negligible to that between alveolar water and air.

Recently, Triphan *et al*. reported a dependence of *T*_1_-relaxation time on the echo time of their inversion recovery snapshot FLASH experiments and pointed out that this requires the presence of two non- or only slow exchange compartments (blood and alveolar water) on the time scale of about one second [55]. The transverse relaxation times expected in lung tissue are around 50 ms (c.f. Fig 4), i.e. about one order of magnitude smaller. Thus, it can be assumed with some certainty that no significant exchange between alveolar magnetization and blood magnetization will occur at the time-scale of *T*_2_ and that both compartments can be treated separately. In our model, the influence of capillary blood movement on Δ*R*_2_ can be accomplished through the incorporation of a pseudo-diffusion coefficient *D*_*p*_ / flow attenuation factor *F* for the blood compartment, as obtained from intravoxel incoherent motion imaging [56]. *D*_*p*_ is about one order of magnitude larger than the self-diffusion coefficient of water, as shown recently for the determination of blood-volume fractions in peripheral lung tissue *in vivo* [57]. In the context of the presented model, this method allows for an adequate separation of the two compartments of blood and tissue and, thus, for a description of the complete magnetization signal as the combination of the signals of alveolar shell and blood shell. In the mean relaxation time approximation, the corresponding relaxation time for the signal will be the weighted sum of the relaxation rates times from each compartment.

It should also be mentioned that determination of alveolar radii from Eq (23) is dependent on a reasonable choice of volume fraction *η* in order to solve the transcendental Eq (17) (typically, *η* ≈ 0.85 [19, 42]). However, numerically incorporating this equation into a multi-parametric fit analysis for both radius and volume fraction proves computationally cumbersome and a rigorous mathematical treatment would go beyond the scope of this work. Another method to determine *η* is available in the form of spectroscopic measurements that quantify the water line-shape in lung tissue. Such experiments date back to Cutillo *et al*. [15] and have recently been re-evaluated by Mulkern *et al*. for Wigner-Seitz foam model geometries [58]. In a similar effort as in [59–62] for Larmor frequency distributions around capillaries (c.f. Fig 3 in [45]), *η* can be extracted from water line-shape measurements as in Fig 3 in [58] or Fig 8 in [63]. Another possibility to acquire *η* is presented by proton density weighted imaging that determines the proton density inside a voxel with a water phantom or adequate macro-vessel as reference, in analogy to the methods presented in [64–66]. Once *η* is determined or reasonably estimated for each voxel-of-interest, a model fit of experimental *R*_2_ values for *τ* (and *R*_2,0_) yields representative radii for each voxel. The problem of averaging over a whole distribution of radii within one imaging voxel is addressed in Appendix C: for large standard deviations of radii within one imaging voxel, a radius correction of about 10% has to be applied. However, while coefficients of variation of radii in comparatively large volumes of peripheral lung tissue are between 0.1–0.5 [19, 42, 51, 67], they are not likely to be very large within one imaging voxel [68].

**Fig 6:**
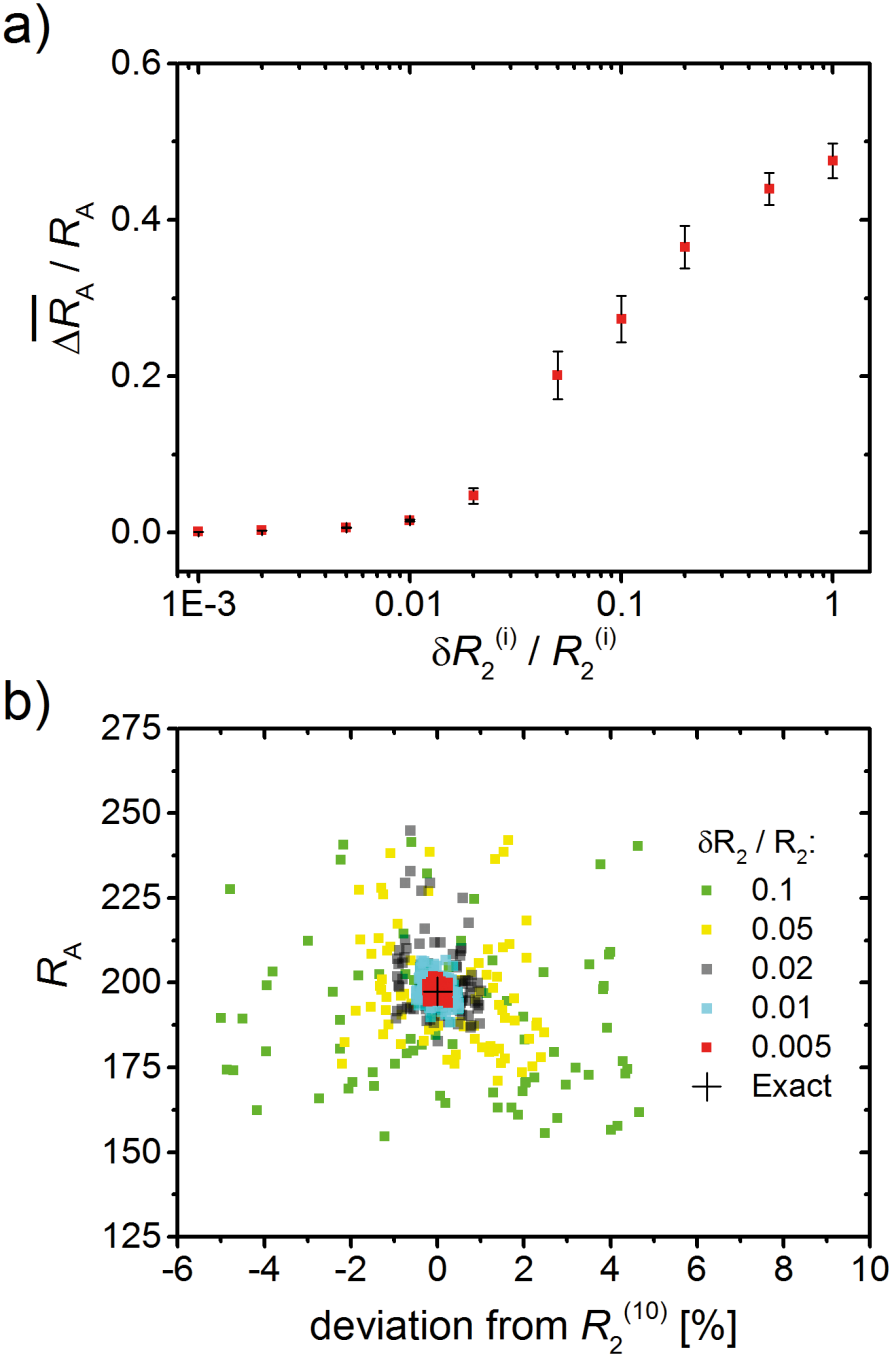
Sensitivity analysis for varying relaxation rates. (a) All measured relaxation rates $R_{2}^{\left(i\right)}$ for the 3.5h experimental data (Fig 5a) were varied within different ranges $\delta R_{2}^{\left(i\right)}$ with $\forall i\text{:}\;\delta R_{2}^{\left(i\right)}/R_{2}^{\left(i\right)}=\text{const}$. For multiple sets of such variations, the average of the resulting difference to and in proportion of the initially obtained radius *R*_A_ is negligible for relative ranges < 0.01. (b) Scatter plot of the resulting radii vs. deviations for the example $R_{2}^{\left(10\right)}=R_{2}\left(\tau_{180}=17\;\text{ms}\right)$ for different strengths of variation; all other $R_{2}^{\left(i\right)}$ values were also varied within their respective error ranges as in (a).

Furthermore, the model regards closely neighboring alveoli as closed entities that have no direct communication as opposed to other respiratory airway models that consider alveoli as forming an alveolar sleeve that originates from a cylindrical airway [12]. However, in the presented model, the effect of large inner and outer surfaces (as compared to the volume of the dephasing volume) has been taken into account with Smoluchowski boundary conditions as detailed above. Also, the model assumes proton diffusion inside the dephasing volume to be barrier-free. This common assumption is standard practice [15, 36] and presumably has only minor effects on the relaxation rate. In addition, the mean linear intercept value for lung tissue has been utilized as the mean alveolar diameter [51] for experimental verification, although some authors argue that the average linear intercept is closer to $\frac{4}{3}R_{\text{A}}$ than 2*R*_A_ [69]. In addition, model sensitivity towards uncertainties in the acquired MR signals was evaluated for the 3.5h hydrogel foam MR data in Fig 5a, see Fig 6. Deviations of model fits for alveolar radii from the initial radius value were found to be negligible for small ranges of variations of measured relaxation rates $\delta R_{2}^{\left(i\right)}/R_{2}^{\left(i\right)}<0.02$.

With a dependence of CPMG relaxation time on local air volume fraction and alveolar radius, the presented model connects parameters that are important for examining and quantifying the pathophysiology of complex lung diseases and general studies of pulmonary ventilation as for example in emphysema.

## Appendix A

The spherical Bessel functions, *j*_2_ and *y*_2_, can be expressed in terms of sine and cosine functions:

$$\begin{array}{c} j_{2}\left(z\right)=[\frac{3}{z^{3}}-\frac{1}{z}]\sin\left(z\right)-\frac{3}{z^{2}}\cos\left(z\right) \end{array}$$ (26)

$$\begin{array}{c} y_{2}\left(z\right)=-[\frac{3}{z^{3}}-\frac{1}{z}]\cos\left(z\right)-\frac{3}{z^{2}}\sin\left(z\right) \end{array}$$ (27)

which allows one to simplify the expansion coefficients to

$$\begin{array}{ccc} G_{n} & = & \frac{24\eta^{\frac{4}{3}}}{5\left[1-\eta \right]\kappa_{n}^{2}}\\ & & \times \;\frac{{\left[\eta^{\frac{2}{3}}[3\kappa_{n}\cos\left(\kappa_{n}\right)+\left[\kappa_{n}^{2}-3\right]\sin\left(\kappa_{n}\right)]+\left[3\eta^{\frac{2}{3}}-\kappa_{n}^{2}\right]\sin\left(\kappa_{n}\eta^{-\frac{1}{3}}\right)-3\eta^{\frac{1}{3}}\kappa_{n}\cos\left(\kappa_{n}\eta^{-\frac{1}{3}}\right)\right]}^{2}}{{\left[3\kappa_{n}\cos\left(\kappa_{n}\right)+\left[\kappa_{n}^{2}-3\right]\sin\left(\kappa_{n}\right)\right]}^{2}-\eta^{\frac{1}{3}}{\left[\left[3\eta^{\frac{2}{3}}-\kappa_{n}^{2}\right]\sin\left(\kappa_{n}\eta^{-\frac{1}{3}}\right)-3\eta^{\frac{1}{3}}\kappa_{n}\cos\left(\kappa_{n}\eta^{-\frac{1}{3}}\right)\right]}^{2}}\;. \end{array}$$ (28)

The following sums, containing eigenvalues *κ*_*n*_ and expansion coefficients *G*_*n*_, are helpful for the subsequent numerical analysis:

$$\begin{array}{ccc} \sum_{n=1}^{\infty }G_{n} & = & \frac{4}{5}\eta \end{array}$$ (29)

$$\begin{array}{ccc} \sum_{n=1}^{\infty }G_{n}\kappa_{n}^{2} & = & \frac{36}{5}\eta \frac{1-\eta^{\frac{5}{3}}}{1-\eta } \end{array}$$ (30)

$$\begin{array}{ccc} \sum_{n=1}^{\infty }\frac{G_{n}}{\kappa_{n}^{2}} & = & \frac{\eta {\left[\eta^{\frac{1}{3}}-1\right]}^{2}\left[4+7\eta^{\frac{1}{3}}+4\eta^{\frac{2}{3}}\right]}{96\left[1+\eta^{\frac{1}{3}}+\eta^{\frac{2}{3}}\right]\left[1+\eta^{\frac{1}{3}}+\eta^{\frac{2}{3}}+\eta +\eta^{\frac{4}{3}}\right]} \end{array}$$ (31)

$$\begin{array}{ccc} \sum_{n=1}^{\infty }\frac{G_{n}}{\kappa_{n}^{4}} & = & \frac{{\left[\eta^{\frac{1}{3}}-1\right]}^{4}\left[1+\eta^{\frac{1}{3}}\right]\eta \left[2+12\eta^{\frac{1}{3}}+17\eta^{\frac{2}{3}}+12\eta +2\eta^{\frac{4}{3}}\right]}{1152\left[1+\eta^{\frac{1}{3}}+\eta^{\frac{2}{3}}\right]{\left[1+\eta^{\frac{1}{3}}+\eta^{\frac{2}{3}}+\eta +\eta^{\frac{4}{3}}\right]}^{2}}\;. \end{array}$$ (32)

The first sums follow from Eqs (1), (7) and (8) (as detailed in the Results section), and the last three sums are calculated as in Appendix B in [29], but for Smoluchowski boundary conditions.

## Appendix B

For large *η* → 1, a Taylor series expansion in [1 − *η*] yields the leading term for *κ*_1_ in Eq (18) as:

$$\begin{array}{c} \kappa_{1}\approx \frac{3\sqrt{6}}{1-\eta }\;. \end{array}$$ (33)

In the same limit, the CPMG transverse relaxation rate can be expressed as

$$\begin{array}{c} \lim_{\eta \to 1}\frac{\Delta R_{2}}{\tau \delta \omega^{2}}=\frac{7}{540}{\left[1-\eta \right]}^{2}[1-\frac{{\left[1-\eta \right]}^{2}\tau }{27\tau_{180}}\text{tanh}(\frac{27\tau_{180}}{{\left[1-\eta \right]}^{2}\tau })]\;. \end{array}$$ (34)

This result coincides with the relaxation rate for two-site chemical exchange in [49] (Eq (3) therein) while the weak magnetization condition *τ*_180_/2 < 1/*δω* is valid [49]. Furthermore, the correlation time in Eq (21) approaches $\frac{\tau }{\kappa_{1}^{2}}=\tau \frac{{\left[1-\eta \right]}^{2}}{54}$. It should be noted that, in such a case, a simple one-dimensional solution for the same absorbing boundary conditions without any gradients produces the same result but would be independent of the alveolar geometry. The one-dimensional case corresponds to diffusion experiments in terms of a Stejskal-Tanner sequence that can be used to quantify the surface relaxivity, as has been previously demonstrated in sedimentary rocks [70].

## Appendix C

The expectation value 〈*R*_A_〉 does not necessarily correspond to $\sqrt{\langle R_{\text{A}}^{2}\rangle }$ as determined through fitting Eq (23) for correlation time *τ*, where $\langle \tau \rangle =\langle R_{\text{A}}^{2}\rangle /D$. It can be evaluated by subtracting the term *δR*_A_ from the fit value $\sqrt{\langle R_{\text{A}}^{2}\rangle }$ with

$$\begin{array}{c} \delta R_{\text{A}}=\sqrt{\left\langle R_{\text{A}}^{2}\right\rangle }[1-\sqrt{1-\frac{\sigma^{2}}{\left\langle R_{\text{A}}^{2}\right\rangle }}] \end{array}$$ (35)

where *σ* represents the standard deviation of the alveolar radii. For a coefficient of variation of 10% for the radii of lung tissue [42], the term *δR*_A_ is negligible since $\delta R_{\text{A}}\approx 0.005\sqrt{\langle R_{\text{A}}^{2}\rangle }$. However, coefficients of variation for lung alveolar radii have been shown in animal and phantom studies to range from 10% to 50% [19, 51, 68, 71]. These variations were detected over large peripheral lung volumes—yet, within the limited collection of alveoli in one typical imaging voxel in a clinical setting with an in-plane resolution of 1.5 × 1.5mm^2^ [72], the variation of alveolar radii is not likely to be very large [68]. Still, alveolar radii in Fig 4c were calculated with a coefficient of variation of 50%, whereas the different *σ* for the radii in Fig 5b were taken as determined in [19] (Fig 12).

To further evaluate changes in the fitting of model radii for uncertainties in the acquired MR signals, random errors from a normal distribution within the interval $\delta R_{2}^{\left(i\right)}$ were added to all measured $R_{2}^{\left(i\right)}$ values for the 3.5h hydrogel foam MR data in Fig 5a (green triangles), see Fig 6, *i* = 1, 2, …, 19 and $\forall i\text{:}\;\delta R_{2}^{\left(i\right)}/R_{2}^{\left(i\right)}=\text{const}$. A full fit of the model for the collection of these varied $R_{2}^{\left(i\right)}$ was then performed to find the deviation Δ*R*_A_ of the resulting radius from the model radius prior to the addition of noise. This procedure was repeated 100 times for different sets of random variations of $R_{2}^{\left(i\right)}$ to find the averaged error $\bar{\Delta R_{\text{A}}}$. The radius error (in units of *R*_A_) is shown in dependence of the applied error ranges for a logarithmic scale in Fig 6a. Evidently, the addition of uncertainty for all $R_{2}^{\left(i\right)}$ values does not change the resulting model radius significantly for ranges $\delta R_{2}^{\left(i\right)}/R_{2}^{\left(i\right)}<0.02$ (see also Fig 6b). This corresponds to a range of 1/50 of the respective *R*_2_ value. For stronger variations with $\delta R_{2}^{\left(i\right)}/R_{2}^{\left(i\right)}>0.05$, the model yields radii that deviate from the initial values by more than 20%. Fig 6b shows a scatter plot of the obtained radii for different deviations (in %) of the example value $R_{2}^{\left(10\right)}=R_{2}\left(\tau_{180}=17\;\text{ms}\right)$ (the initial value is: $R_{2}^{\left(10\right)}=2.8\;{\text{s}}^{-1}$) while all other $R_{2}^{\left(i\right)}$ are also varied within their respective error ranges $\delta R_{2}^{\left(i\right)}$. Except for some outliers in the range of 0.02, most obtained radii are close to the exact value. Though, for a range of 0.1, the obtained radii show a wide spread around the exact value. However, *R*_2_ deviations in ranges ≤0.01 might be achieved with an SNR of 200–1000 for lung imaging at 0.1T, and stronger fields *B*_0_ > 0.8T should increase SNR [73].
